# Unintended pregnancy, contraceptive use, and childbearing desires among HIV-infected and HIV-uninfected women in Botswana: across-sectional study

**DOI:** 10.1186/s12889-015-2498-3

**Published:** 2016-01-16

**Authors:** Gloria K. Mayondi, Kathleen Wirth, Chelsea Morroni, Sikhulile Moyo, Gbolahan Ajibola, Modiegi Diseko, Maureen Sakoi, Jane Dipuo Magetse, Kebaiphe Moabi, Jean Leidner, Joseph Makhema, Betsy Kammerer, Shahin Lockman

**Affiliations:** 1 Botswana-Harvard AIDS Institute Partnership, Private Bag Bo 320, Gaborone, Botswana; 2 Harvard School of Public Health, Boston, MA USA; 3 EGA Institute for Women’s Health/Institute for Global Health, University College London, 74 Huntley Street, WC1E 6AU, London, UK; 4 University of Botswana, Department of Public Health Medicine, University of Botswana Main Campus, Block 246, Gaborone, Botswana; 5 Botswana-UPenn Partnership, University of Botswana Main Campus, 244G Gaborone, Botswana; 6 Goodtables Data Consulting, 3101 Tisbury Rd., Norman, 73071 OK USA; 7 Boston Children’s Hospital, Boston, MA USA; 8 Harvard Medical School, Boston, MA USA; 9 Brigham and Women’s Hospital, 15 Francis Street, PBB 4A, Boston, 02115 MA USA

**Keywords:** Unintended pregnancy, Contraception, Family planning, HIV, Africa, Botswana, Tshipidi

## Abstract

**Background:**

Little is known about the impact of knowledge of HIV serostatus on pregnancy intention and contraceptive use in high-HIV-burden southern African settings in the era of widespread antiretroviral treatment availability.

**Methods:**

We analyzed interview data collected among 473 HIV-uninfected and 468 HIV-infected pregnant and recently postpartum women at two sites in southern Botswana. Participants were interviewed about their knowledge of their HIV status prior to pregnancy, intendedness of the pregnancy, contraceptive use, and future childbearing desires.

**Results:**

The median age of the 941 women was 27 years, median lifetime pregnancies was 2, and 416 (44 %) of pregnancies were unintended. Among women reporting unintended pregnancy, 36 % were not using a contraceptive method prior to conception. Among contraception users, 81 % used condoms, 13 % oral contraceptives and 5 % an injectable contraceptive. In univariable analysis, women with unintended pregnancy had a higher number of previous pregnancies (*P* = <0.0001), were less educated (*P* = 0.0002), and less likely to be married or living with a partner (*P* < 0.0001). Thirty-percent reported knowing that they were HIV-infected, 48 % reported knowing they were HIV-uninfected, and 22 % reported not knowing their HIV status prior to conception. In multivariable analysis, women who did not know their HIV status pre-conception were more likely to report their pregnancy as unintended compared to women who knew that they were HIV-uninfected (aOR = 1.7; 95%CI: 1.2-2.5). After controlling for other factors, unintended pregnancy was not associated with knowing one’s HIV positive status prior to conception (compared with knowing one’s negative HIV status prior to conception). Among women with unintended pregnancy, there was no association between knowing their HIV status and contraceptive use prior to pregnancy in adjusted analyses. Sixty-one percent of women reported not wanting any more children after this pregnancy, with HIV-infected women significantly more likely to report not wanting any more children compared to HIV-uninfected women (aOR = 3.9; 95%CI: 2.6-5.8).

**Conclusions:**

The high rates of reported unintended pregnancy and contraceptive failure/misuse underscore an urgent need for better access to effective contraceptive methods for HIV-uninfected and HIV -infected women in Botswana. Lower socioeconomic status and lack of pre-conception HIV testing may indicate higher risk for unintended pregnancy in this setting.

## Background

Sub-Saharan Africa continues to have the highest rates of new HIV infections [1] as well as unintended pregnancies [2]. As a region, sub-Saharan Africa has the lowest level of contraception prevalence [3] with an estimated 25 % unmet need for family planning among women aged 15 and 49 [4]. Eliminating unmet need for effective family planning among all women has great personal, familial and societal benefits. This includes reduced maternal and child mortality and better educational and economic gender equity [5–7]. In addition, the World Health Organization mentions reducing the unmet need for family planning among women living with HIV as a highly cost-effective strategy that is essential for the elimination of MTCT [7, 8].

Improving family planning use requires the availability of contraceptive methods that allow women to align their choice of method with their reproductive intentions. Although short-acting methods such as condoms, oral contraceptives (OCs) and injectables are the most common methods in sub-Saharan Africa, long-acting reversible (LARC) and permanent methods are more effective under “typical use” conditions, as they eliminate the need for adherence and have higher continuation rates [9, 10]. Hence, unintended pregnancy could be high in Botswana, where the available methods are mostly short-acting, requiring daily or quarterly adherence. The current use of modern contraception among women 15–49 in Botswana has increased from 16 % in 1984 to 51 % in 2007. The most commonly used contraceptives are condoms, oral and injectable contraceptives; use of LARC methods such as the intrauterine device and implants is negligible [11]. The male condom comprised 93.3 % of current use in 2006 [12], and the condom’s predominance has been attributed to a national multimedia Condom Social Marketing Program implemented by the Botswana Population Services in 1994 and 1995 in response to the HIV epidemic [11]. In Botswana, where 30.4 % of women of reproductive age are HIV-infected [13] and antiretroviral treatment (ART) coverage is high [14, 15], access to a variety of contraceptive options is critical because HIV-infected women may have particular contraceptive needs and may change their childbearing plans in response to learning their HIV serostatus [16, 17].

We assessed pregnancy intention, contraceptive use, and future childbearing desires among pregnant and recently post-partum HIV-positive and HIV-negative women in Botswana. Our objectives were to: 1) assess the frequency of unintended pregnancy among women according to knowledge of HIV serostatus prior to becoming pregnant; 2) determine whether prior knowledge of HIV serostatus is an independent predictor of unintended pregnancy; 3) determine among women with unintended pregnancies, whether prior knowledge of HIV serostatus is associated with contraceptive use; and 4) compare future childbearing desires by actual HIV serostatus at study enrolment. There are currently no published data on these topics from Botswana and little data from southern Africa in this era of widespread ART use.

## Methods

### Study population

This was a planned analysis of baseline data collected in a prospective observational cohort study of child health and neurodevelopment (“Tshipidi” study: a Setswana word meaning “a journey of a thousand miles begins with one step”). This study was funded by the National Institute of Mental Health at the National Institutes of Health, R01MH087344. In total, 475 HIV-uninfected and 474 HIV-infected pregnant and recently postpartum women aged 18 years and older and their infants were enrolled between 2010 and 2012 at 28 antenatal clinics and 5 maternity wards in two locations in Botswana: the capital city Gaborone and in the large village of Mochudi, which is 45 km North of Gaborone. As part of the main cohort study, mothers and infants were followed for two years to evaluate the effect of maternal HIV status on child health and neurodevelopmental outcomes. Participants were enrolled antepartum (*n* = 805 (84.8 %)) or within seven days of delivery (*n* = 144 (15.2 %)). The current analysis utilizes data from structured questionnaires administered privately at enrollment by trained study nurses at the time of enrollment.

### Outcome assessment

To measure pregnancy intendedness, the primary outcome, all women were asked “Were you trying to become pregnant when you conceived the baby?” This yielded a combined category of unintended pregnancy, which included mistimed and unwanted pregnancies, irrespective of whether contraception was being used. Women who reported that they were not trying to become pregnant at the time of conception were also asked whether they were using contraception and the specific contraceptive method type was documented. We considered women as having experienced contraceptive failure or misuse (including poor adherence), if they reported that the current pregnancy was unintended and that they were using contraception prior to the pregnancy. Women were considered as having an unmet need for family planning if they reported that the current pregnancy was unintended and that they were not using contraception prior to the pregnancy.

### Exposure assessment

Women’s self-reported knowledge of HIV status prior to becoming pregnant was recorded as “known HIV-infected”, “known HIV-uninfected” or “unknown HIV status.” HIV status at the time of study enrolment was also determined by confirmatory testing on all participants.

### Statistical analysis

We performed basic descriptive analyses, including a comparison of study enrolment characteristics stratified by maternal knowledge of HIV serostatus prior to becoming pregnant, and intendedness of pregnancy. We used logistic regression to estimate univariable and multivariable-adjusted odds ratios (OR) and 95 % confidence intervals (CI) for pregnancy intendedness. We considered several covariates that likely temporally preceded the current pregnancy and birth and might also be associated with knowledge of HIV status prior to becoming pregnant and pregnancy intendedness. Therefore, we included all of the following variables in the multivariable-adjusted model as potential confounders: enrollment site, age, relationship status, educational level, employment status, income, household size, household assets, and lifetime number of pregnancies and history of a child dying after birth but before five years of age. All statistical analyses were conducted using SAS software version 9.3 (SAS Institute, Cary, NC).

In secondary analyses, we used the same process to assess the relationship between a) maternal knowledge of HIV serostatus prior to becoming pregnant and contraceptive use and b) HIV status at enrollment and future childbearing desires in multivariate-adjusted analyses.

### Ethics

Ethics approval for the Tshipidi study was obtained from the Office of Human Research Administration (OHRA) at the Harvard School of Public Health and the Health Research Development Committee at the Botswana Ministry of Health. All women provided written informed consent prior to study participation. Only unique study identifiers were used in the database.

## Results

### Descriptive characteristics

Nine hundred and forty-nine women were enrolled in the Tshipidi study, and 941 had complete data for this analysis. The median age of the participants was 27 years, 32 % were married or living with a partner, and more than half were unemployed (57 %) and had no income (61 %). They had a median of 2 lifetime pregnancies (range1-9). Table 1 presents socio-demographic and reproductive characteristics of participants stratified by knowledge of HIV serostatus prior to the current pregnancy.

**Table 1:** Baseline characteristics of N = 941 pregnant and postpartum adult women enrolled in a prospective observational cohort study in Botswana, 2010–2012 overall and according to knowledge of HIV status prior to becoming pregnant

		Maternal knowledge of HIV serostatus prior to becoming pregnant			
Characteristic (total *n* with data)	Total (N = 941)	Knew she was HIV-infected (N = 288)^a^	Knew she was HIV-uninfected (N = 446)^a^	Did not know her HIV status (N = 207)	P*
Demographics					
Location (*n* = 941)					0.16
Mochudi	394 (42 %)	117 (41 %)	200 (45 %)	77 (37 %)	
Gaborone	547 (58 %)	171 (59 %)	246 (55 %)	130 (63 %)	
Age, in years (*n* = 941)					<0.0001
18 to 24 years	319 (34 %)	46 (16 %)	169 (38 %)	104 (50 %)	
25 to 29 years	261 (28 %)	69 (24 %)	143 (32 %)	49 (24 %)	
30 to 34 years	216 (23 %)	98 (34 %)	87 (20 %)	31 (15 %)	
25 to 39 years	105 (11 %)	56 (19 %)	33 (7 %)	16 (8 %)	
40 years and older	40 (4 %)	19 (7 %)	14 (3 %)	7 (3 %)	
Median (IQR)^b^	27 (23, 32)	30 (26, 34)	26 (22, 30)	24 (21, 30)	<0.0001**
Relationship status (*n* = 941)					0.10
Married	70 (7 %)	24 (8 %)	35 (8 %)	11 (5 %)	
Not married, but living together	239 (25 %)	75 (26 %)	116 (26 %)	48 (23 %)	
Not married and not living together	574 (61 %)	167 (58 %)	278 (62 %)	129 (62 %)	
Not in a serious relationship	58 (6 %)	22 (8 %)	17 (4 %)	19 (9 %)	
Maternal HIV serostatus at enrolment (*n* = 941)					<0.0001
HIV-uninfected	473 (50 %)	9 (3 %)	377 (85 %)	87 (42 %)	
HIV-infected	468 (50 %)	279 (97 %)	69 (15 %)	120 (58 %)	
Socioeconomic factors					
Education (*n* = 939)					<0.0001
None or primary	94 (10 %)	49 (17 %)	24 (5 %)	21 (10 %)	
Junior secondary	449 (48 %)	176 (61 %)	178 (40 %)	95 (46 %)	
Senior secondary	272 (29 %)	43 (15 %)	162 (36 %)	67 (32 %)	
Tertiary	124 (13 %)	19 (7 %)	81 (18 %)	24 (12 %)	
Employment (*n* = 939)					0.20
Employed	401 (43 %)	130 (45 %)	188 (42 %)	83 (40 %)	
Unemployed and looking for work	248 (26 %)	80 (28 %)	121 (27 %)	47 (23 %)	
Unemployed but not looking for work^c^	290 (31 %)	77 (27 %)	137 (31 %)	76 (37 %)	
Income (*n* = 939)^d^					<0.0001
None	574 (61 %)	161 (56 %)	289 (65 %)	124 (60 %)	
<$57 per month	50 (5 %)	28 (10 %)	13 (3 %)	9 (4 %)	
$57 to $113 per month	144 (15 %)	53 (19 %)	48 (11 %)	43 (21 %)	
>$113 per month	171 (18 %)	44 (15 %)	96 (22 %)	31 (15 %)	
Household size (*n* = 938)					0.04
0 to 4 people	277 (30 %)	83 (29 %)	139 (31 %)	55 (27 %)	
5 to 6 people	201 (21 %)	54 (19 %)	107 (24 %)	40 (19 %)	
7 to 9 people	218 (23 %)	80 (28 %)	95 (21 %)	43 (21 %)	
10 or more people	242 (26 %)	70 (24 %)	104 (23 %)	68 (33 %)	
Television or car in the household (*n* = 935)	619 (66 %)	168 (59 %)	325 (73 %)	126 (61 %)	0.0001
Reproductive history					
Total number of prior pregnancies including current pregnancy (*n* = 941)					<0.0001
1	296 (31 %)	34 (12 %)	165 (37 %)	97 (47 %)	
2	279 (30 %)	77 (27 %)	159 (36 %)	43 (21 %)	
3	175 (19 %)	74 (26 %)	62 (14 %)	39 (19 %)	
4 or more	191 (20 %)	103 (36 %)	60 (13 %)	28 (14 %)	
Median (IQR)^b^	2 (1 – 3)	3 (2 – 4)	2 (1 – 3)	2 (1 – 3)	<0.0001**
Any child died before age 5 years (*n* = 941)	93 (10 %)	51 (18 %)	26 (6 %)	16 (8 %)	<0.0001
Current pregnancy unintended (*n* = 940)	416 (44 %)	141 (49 %)	168 (38 %)	107 (52 %)	0.001
Contraception use prior to current pregnancy among women with unintended pregnancy (*n* = 416)					0.54
Was using contraception	268 (64 %)	93 (66 %)	103 (61 %)	72 (67 %)	
Was not using contraception	148 (36 %)	48 (35 %)	65 (39 %)	35 (33 %)	
Type of contraception used (*n* = 269)					0.41
Oral contraceptive pills	36 (13 %)	16 (17 %)	11 (11 %)	9 (13 %)	
Injectable contraception	14 (5 %)	4 (4 %)	8 (8 %)	2 (3 %)	
Condoms	219 (81 %)	74 (79 %)	84 (82 %)	61 (85 %)	
Intrauterine device, diaphragm or other	0 (0 %)	0 (0 %)	0 (0 %)	0 (0 %)	
Future desire for children (*n* = 941)					<0.0001
No	577 (61 %)	223 (77 %)	225 (50 %)	129 (62 %)	
Yes	229 (24 %)	34 (12 %)	155 (35 %)	40 (19 %)	
Do not know	135 (14 %)	31 (11 %)	66 (15 %)	38 (18 %)	

*IQR* interquartile range

*Wald χ^2^ *P*-values unless otherwise noted

**Wilcoxon rank sum *P*-values

^a^Data are n (column %) unless otherwise noted. Values may not total to 100 % due to rounding

^b^25^th^ and 75^th^ percentiles

^c^Unemployed includes participants reporting housewife as primary occupation

^d^Botswana pula were converted to US dollars based on a rate of 8.87 pula per US$1

Of the 941 pregnancies, 416 (44 %) were reported as unintended (Table 1). Among the 416 women who reported that the current pregnancy was unintended, 268 (64 %) reported using a contraceptive method around the time of conception; these women presumably experienced contraceptive failure or did not use contraceptives correctly (misuse). The remaining 148 (36 %) women with an unintended pregnancy reported not using a contraceptive method around the time of conception; these women had an unmet need for family planning. Of the 268 women who reported contraceptive use and not intending to become pregnant (i.e., possible contraceptive failure/misuse), 81 % (*N* = 219) were using male condoms, 13 % (*N* = 36) were using oral contraceptives and 5 % (*N* = 14) were using DMPA injectable contraceptive. None were using long-acting reversible (LARC) or permanent methods.

### Factors associated with unintended pregnancy

In univariable analyses, compared to women with intended pregnancies, women with unintended pregnancies were less educated (*P* = 0.0002), more likely to be unemployed (*P* = 0.004), more likely not to be in a serious relationship (*P* = <0.0001), more likely to have had more previous pregnancies (*P* = <0.0001), more likely to have larger households (*P* = 0.02), and less likely to have a television or car in the household (*P* = 0.01) (Table 2).

**Table 2:** Univariable and multivariable-adjusted analysis of factors associated with current pregnancy reported being unintended among pregnant and postpartum adult women enrolled in a prospective observational cohort study in Botswana, 2010-2012

	Current pregnancy unintended		Univariable Models			Full Multivariable Model		
	N	(%)^a^	OR	(95 % CI)	P*	OR	(95 % CI)	P*
Exposure of interest								
Maternal knowledge of HIV status prior to becoming pregnant					0.01			0.02
Known to be HIV-uninfected	168	(40 %)	1	(ref)		1	(ref)	
HIV status unknown	107	(26 %)	1.77	(1.27, 2.47)		1.71	(1.18, 2.47)	
Known to be HIV-infected	141	(34 %)	1.60	(1.18, 2.16)		1.30	(0.91, 1.86)	
Demographics								
Location					0.80			0.89
Mochudi	172	(41 %)	0.97	(0.74, 1.26)		0.98	(0.71, 1.35)	
Gaborone	244	(59 %)	1	(ref)		1	(ref)	
Age, in years					0.07			0.02
18 to 24 years	146	(35 %)	1	(ref)		1	(ref)	
25 to 29 years	104	(25 %)	0.79	(0.65, 1.09)		0.62	(0.41, 0.94)	
30 to 34 years	88	(21 %)	0.82	(0.58, 1.17)		0.43	(0.26, 0.70)	
25 to 39 years	56	(13 %)	1.35	(0.87, 2.11)		0.57	(0.31, 1.06)	
40 years and older	22	(5 %)	1.45	(0.75, 2.80)		0.42	(0.18, 1.00)	
Relationship status					<0.0001			<0.0001
Married	25	(6 %)	0.76	(0.45, 1.27)		0.71	(0.39, 1.28)	
Not in a serious relationship	48	(12 %)	6.57	(3.26, 13.24)		7.18	(3.31, 15.59)	
Not married, but living together	101	(24 %)	1.00	(0.74, 1.36)		0.90	(0.63, 1.28)	
Not married and not living together	242	(58 %)	1	(ref)		1	(ref)	
Socioeconomic factors								
Education					0.0002			0.22
None or primary	60	(14 %)	1	(ref)		1	(ref)	
Junior secondary	196	(47 %)	0.44	(0.28, 0.70)		0.60	(0.35, 1.01)	
Senior secondary	116	(28 %)	0.42	(0.26, 0.68)		0.72	(0.40, 1.31)	
Tertiary	43	(10 %)	0.30	(0.17, 0.53)		0.61	(0.31, 1.21)	
Employment					0.004			0.003
Employed	168	(40 %)	1	(ref)		1	(ref)	
Unemployed and looking for work	132	(32 %)	1.58	(1.15, 2.17)		1.02	(0.58, 1.79)	
Unemployed but not looking for work	116	(28 %)	0.93	(0.68, 1.27)		0.54	(0.31, 0.95)	
Income^b^					0.15			0.24
None	264	(63 %)	1	(ref)		1	(ref)	
<$57 per month	27	(6 %)	1.37	(0.77, 2.45)		0.81	(0.39, 1.67)	
>$113 per month	58	(14 %)	0.75	(0.53, 1.07)		0.72	(0.39, 1.32)	
$57 to $113 per month	67	(16 %)	0.79	(0.55, 1.14)		0.52	(0.27, 0.99)	
Household size					0.02			0.05
0 to 4 people	101	(24 %)	1	(ref)		1	(ref)	
5 to 6 people	92	(22 %)	1.47	(1.02, 2.13)		1.59	(1.07, 2.36)	
7 to 9 people	102	(25 %)	1.55	(1.08, 2.22)		1.62	(1.07, 2.45)	
10 or more people	119	(29 %)	1.69	(1.19, 2.40)		1.52	(1.02, 2.29)	
Television or car in the household								
No	159	(39 %)	0.68	(0.52, 0.90)	0.01	0.85	(0.63, 1.16)	0.31
Yes	253	(61 %)	1	(ref)		1	(ref)	
Reproductive history								
Total number of prior pregnancies including current pregnancy					<0.0001			<0.0001
1	110	(26 %)	1	(ref)		1	(ref)	
2	103	(25 %)	1.00	(0.71, 1.40)		1.56	(1.04, 2.34)	
3	91	(22 %)	1.83	(1.25, 2.68)		3.45	(2.07, 5.75)	
4 or more	112	(27 %)	2.40	(1.65, 3.48)		4.76	(2.62, 8.65)	
Any child died before age 5 years	41	(10 %)	0.99	(0.65, 1.53)	0.97	0.57	(0.34, 0.94)	0.03

*OR* odds ratio, *CI* confidence interval

*Global Wald *χ*2 *P*-values

^a^Column percentage (values may not sum to 100 % due to rounding)

^b^Botswana pula were converted to US dollars based on a rate of 8.87 pula per US$1

### Prior knowledge of HIV status and unintended pregnancy

Most women (78 %) reported knowing their HIV status prior to conception of their pregnancy; 30 % knew that they were HIV-infected, 48 % knew that they were HIV-uninfected and 22 % reported not knowing their HIV status (Table 1). In univariable analysis, women who knew that they were HIV-infected or who did not know their HIV serostatus pre-pregnancy were more likely to have had an unintended pregnancy than women who knew that they were HIV-uninfected. In multivariable analysis (Table 2), women who reported not knowing their HIV serostatus prior to becoming pregnant were 1.7 times more likely (95 % CI:1.2-2.5) to report their pregnancy as unintended compared to women who knew that they were HIV-uninfected prior to becoming pregnant. However, knowledge of positive HIV serostatus prior to conception was no longer significantly associated with unintended pregnancy after controlling for other factors (aOR compared with knowledge of negative serostatus: 1.3; 95 % CI: 0.9-1.9). Other factors independently associated with reporting an unintended pregnancy were age, not being in a serious relationship, larger household size, and having had more previous pregnancies. Unintended pregnancy was less likely to be reported among women who had a child die before the age of 5 years.

### Prior knowledge of HIV serostatus and contraceptive use

Among the 416 women with an unintended pregnancy, levels of contraceptive use did not differ by knowledge of HIV serostatus prior to becoming pregnant: 66 % (*N* = 93) of known HIV-infected women reported contraceptive use compared to 61 % (*N* = 103) of known HIV-uninfected and 67 % (*N* = 72) of those with unknown HIV status (*P* = 0.54). There were no significant differences in the types of contraception reported across these three groups (*P* = 0.4), with the vast majority in all groups using the male condom. Adjustment for socio-demographics and reproductive characteristics in multivariable analysis did not change this result; women who knew they were HIV-infected and women who did not know their HIV status were just as likely to report contraceptive use as women who knew they were HIV uninfected, indicating that knowledge of HIV serostatus prior to becoming pregnant was not independently associated with contraceptive use.

### HIV serostatus at enrolment and future childbearing desires

At enrolment, 468 women were confirmed to be HIV-infected. Of these women, 279 (60 %) reported being aware of their HIV-positive serostatus before the pregnancy and the remaining 189 (40 %) reported being first diagnosed with HIV during or after pregnancy. Sixty-one percent of women reported wanting no more children after this pregnancy. We observed a significant difference in future childbearing desires by actual HIV serostatus: 46 % of HIV-uninfected women compared to 76 % of HIV-infected women did not want more children in the future (*P* < 0.0001). In multivariable analysis adjusted for socio-demographic characteristics and reproductive history (data not shown), HIV-infected women were almost four times more likely to report not wanting *any more* children compared to HIV-uninfected women (OR: 3.9; 95 % CI: 2.6-5.8). Other factors independently associated with not wanting more children in the future were greater numbers of previous pregnancies (*P* < 0.0001) and not having had a child die before the age of 5 years (*P* < 0.0001).

## Discussion

This is the first published data on unintended pregnancy, contraceptive use and HIV serostatus in Botswana, a country where 30.4 % of women of reproductive age are HIV-infected [13]. We found that both contraceptive failure (or misuse) and unmet need for family planning may have contributed to the high levels of unintended pregnancies among both HIV-infected and HIV-uninfected women, similar to findings from other recent studies in sub-Saharan Africa [10, 18]. Overall, 44 % of women from two areas of Botswana reported that their pregnancy was unintended. The Botswana 2013 Global AIDS Response Report estimated levels of unplanned pregnancy at 50 % [19]. Factors associated with unintended pregnancy in our study were similar to those found in other studies in sub-Saharan Africa [10, 20].

Reported contraceptive use prior to unintended pregnancy was high (64 %), but the methods used were exclusively short-acting requiring daily (OC), quarterly adherence (DMPA), or with every act of sexual intercourse (male condom). The vast majority of women with an unintended pregnancy (81 %), regardless of HIV serostatus, reported relying on the male condom for contraception. Our findings suggest that reliance on condoms for pregnancy prevention is not an effective strategy. This is supported by recent studies in areas of high HIV prevalence, which have shown that more effective methods of contraception are substituted for male condom-only use [20–22]. Women and couples should be informed that while male condoms are the only contraceptive method that can reduce the risk of sexually transmitted infections including HIV and that their correct and consistent use is imperative in that regard, they may have a high failure rate for pregnancy prevention. This is likely due to low levels of correct and consistent use, with a typical-use contraceptive failure rate for the male condom of about 21 % within the first year [23]. Ideally in the context of high levels of HIV infection, a dual method approach (i.e., combining condoms, male or female, with a highly effective contraceptive method) should be promoted [4].

Our data highlight the urgent need for women’s access to effective contraceptive methods that align with pregnancy intentions and reduce the potential for incorrect or inconsistent use. LARCs such as the intrauterine device (IUD) and the contraceptive implant reduce or eliminate the need for daily or per-act of intercourse adherence. The IUD is not frequently provided in Botswana’s public health sector, with its use among women aged 12–49 having peaked at 4.1 % in the late 1980’s and declining since to about 0.8 % in 2007, due to now disproved safety concerns about IUD use in the context of HIV [24]. Sterilization has never been widely practiced in Botswana (utilized among <2 % of women aged 12–49 from 1985 to 2007) [24]. Fortunately, the Botswana Sexual and Reproductive Health Department has recently prioritized increasing the supply of and demand for the LARC methods (personal communication, Sexual and Reproductive Health Division, Botswana Ministry of Health).

Further objectives of our study were to examine heterogeneity in unintended pregnancy, contraceptive use and future childbearing desires by HIV serostatus. We found that 26 % of HIV-infected women reported not knowing their HIV status prior to conception. While it is possible that some of these women did in fact know that they were HIV-infected but were not comfortable disclosing this at the time of study enrollment, this may also suggest missed opportunities for HIV diagnosis prior to pregnancy, and thus missed opportunities for pregnancy planning and safer conception or contraceptive counseling for HIV-infected women. Sixty-nine women (15 %) who reported known HIV-uninfected status before pregnancy were diagnosed with HIV during their pregnancy, possibly reflecting a high risk of seroconversion in pregnancy than has been previously reported in Botswana [25] or elsewhere (some of these women may also have known that they were HIV-infected pre-conception but not disclosed this at study enrollment, as noted above). These findings support the need for continuing HIV-prevention efforts before and during pregnancy.

Levels of unintended pregnancy were high irrespective of knowledge of HIV serostatus prior to becoming pregnant. The prevalence of unintended pregnancy was significantly higher among women who reported not knowing their HIV status (52 %) compared to those who knew they were HIV-uninfected (38 %) even after controlling for other factors. Considering that HIV-infected women generally maintain regular contact with health services and may have strong motivations to prevent pregnancy, it is discouraging that unmet needs for effective family planning were high. That women of unknown HIV status report the highest levels of unintended pregnancy is not surprising given that these women may be the least engaged with health services of any kind.

Levels of contraceptive use prior to the unintended pregnancy and method type did not differ by knowledge of HIV serostatus prior to becoming pregnant. This contrasts with recent data from Zambia [26] where contraceptive use at time of conception was higher in HIV-infected women than their HIV uninfected counterparts. Other studies from sub-Saharan Africa have shown that HIV-infected women may have poorer access to contraception than HIV-uninfected women, potentially due to barriers accessing family planning services or fear of stigma from providers [27–29]. Previous studies from Southern Africa have found that safe and effective long-acting methods are sometimes not recommended by providers or accessible to women living with HIV due to limited knowledge and skills of the health care workers providing HIV services; lack of operational guidelines; and poorly integrated reproductive health/family planning and HIV services [22, 30–32]. Historically, HIV and family planning services have been provided separately in Botswana. There are current efforts by the Botswana Ministry of Health to integrate and link strategies across health services [33]. Our data suggest that in Botswana the challenges to obtaining effective contraception may apply relatively equally to both HIV-uninfected and HIV-infected women.

Most participants (61 %) reported not wanting additional children in the future, and this was strongly and independently associated with being HIV-infected, though it is important to note that a large proportion of HIV uninfected women also reported not wanting more children. Unfortunately, we did not collect data on post-partum contraceptive counseling or use, and thus cannot comment on how women’s post-partum contraceptive use will or will not allow them to meet their stated future childbearing desires. However, the methods most widely available and promoted in Botswana at present (male condoms and OCs) are unlikely to enable many of these women to achieve their stated desires. Long-acting reversible and permanent methods are considered the most appropriate for women who do not want more children [34, 35].

It is important to note that while this study was focused on unintended pregnancy, 51 % of pregnancies were intended among women who knew that they were HIV-infected at the time of conception. When asked about future childbearing plans, 23 % of HIV-infected women reported wanting (or not knowing if they wanted) more children in the future. Research indicates that health care providers seldom discuss family planning with their HIV-infected clients [36]. Nevertheless, accumulating data, including these data from Botswana, indicate that HIV-infected women continue to seek and achieve pregnancy following diagnosis with HIV infection [17, 36–38]. Supportive, informed patient–provider communication about fertility intentions and safer conception is essential in this context.

This study has limitations. We assessed HIV status at the time of conception by self-report and did not have data on the timing of the most recent HIV test. Our pregnancy intention measure was a simplification of commonly used measures, and did not distinguish unwanted versus mistimed pregnancies, limiting direct comparison with some other studies. Reporting of pregnancy intention may have been subject to reporting bias, especially if HIV-infected women are differentially counseled to delay or avoid childbearing and over-report unintended pregnancy, and the reporting of condom use by known HIV positive women may be subject to social desirability bias. Furthermore, in our analysis of factors associated with pregnancy intention there may be unmeasured confounders that would bias our estimates of independent effect, which must be considered when interpreting the results. Finally, we did not collect information on desire for contraception prior to conception or post -partum.

## Conclusion

We observed high rates of self-reported unintended pregnancy, and of lack of contraceptive use (or contraceptive failure/misuse), among both HIV-infected and HIV -uninfected women in Botswana. Our results underscore the need to improve access to effective family planning for all women who do not wish to become pregnant at the time, including HIV-infected women, in Botswana, and to promote long-acting reversible and dual contraceptive method use strategies among women who require condoms for HIV/STI prevention. It also highlights the need to explore whether and how healthcare providers in Botswana are supporting and counseling HIV-infected women who intend to conceive.
